# Comparative effectiveness of interventions for preventing tuberculosis: systematic review and network meta-analysis of interventional studies

**DOI:** 10.1016/j.eclinm.2023.102209

**Published:** 2023-09-09

**Authors:** Alemneh Mekuriaw Liyew, Beth Gilmour, Archie C.A. Clements, Kefyalew Addis Alene

**Affiliations:** a Institute of Public Health, College of Medicine and Health Sciences, University of Gondar, Gondar, Ethiopia; b School of Population Health, Faculty of Health Sciences, Curtin University, Australia; c Geospatial and Tuberculosis Research Team, Telethon Kids Institute, Australia; d Peninsula Medical School, University of Plymouth, Plymouth, United Kingdom

**Keywords:** Effectiveness, Network meta-analysis, Preventive interventions, Tuberculosis

## Abstract

**Background:**

Tuberculosis (TB) is the leading infectious cause of death globally. Several preventive measures are employed to prevent TB, yet there is a paucity of evidence on the effectiveness of these interventions. Therefore, this study aimed to identify the most effective interventions for reducing TB incidence.

**Methods:**

A systematic search was undertaken across five relevant databases including PubMed, SCOPUS, Web of Science, Cochrane Central Register of Controlled Trials, and ClinicalTrials.gov from inception to February 22, 2023. Bayesian network meta-analysis (NMA) was conducted to compare the effectiveness of preventive interventions including preventive therapy, nutritional intervention, targeted screening, and vaccination in reducing TB incidence. Subgroup analysis was conducted to investigate the effectiveness of TB preventive treatments.

**Findings:**

Overall 82 articles were included in the NMA. Preventive therapy (OR = 0.44, 95% CrI 0.36–0.52), BCG vaccination (OR = 0.62, 95% CrI 0.39–0.98) and TB candidate vaccines (OR = 0.67, 95% CrI 0.46–0.98) were more effective than placebo or no intervention. When all active interventions were considered, preventive therapy ranked as the best intervention. Of the preventive treatments, isoniazid (OR = 0.46, 95% CrI 0.35–0.55), isoniazid plus rifampicin (OR = 0.56, 95% CrI 0.32–0.97), isoniazid plus rifapentine (OR = 0.49, 95% CrI 0.29–0.83), isoniazid plus ethambutol (OR = 0.39, 95% CrI 0.15–0.99), isoniazid plus streptomycin (OR = 0.12, 95% CrI 0.02–0.55), rifampicin (OR = 0.41, 95% CrI 0.18–0.92), and rifampicin plus pyrazinamide (OR = 0.51, 95% CrI 0.29–0.87) surpassed placebo/none.

**Interpretation:**

Our study suggested that when all available preventive interventions are considered, preventive therapy is likely the most effective intervention. Within TB preventive treatments, isoniazid plus streptomycin is likely ranked at the top. This comparative study provides important information for policymakers and stakeholders, enabling them to make informed decisions on preventive strategies, whilst considering local resources and capacity constraints.

**Funding:**

10.13039/501100001797Curtin University strategic scholarship and Australian National Health and Medical Research Council, through an Emerging Leadership Investigator grant.

**Research in context**

**Evidence before this study**

Several public health interventions including preventive therapy, nutritional therapy, vaccination, and targeted screening are available for the prevention and control of TB. However, their comparative effectiveness in reducing TB incidence has yet to be investigated. Based on a systematic search on February 20, 2021, in PubMed using the search terms “tuberculosis” and “network meta-analysis,” we found 20 network meta-analyses related to various aspects of TB, including TB treatment regimens, latent TB infection (LTBI), TB adverse events, and TB diagnostic methods. However, none of these network meta-analyses specifically investigated the comparative effectiveness of interventions in reducing TB incidence.

**Added value of this study**

To our knowledge, our study provides the first comprehensive systematic review and network meta-analysis of all available evidence comparing TB interventions with placebo or no interventions in reducing the incidence of TB. Our findings showed that preventive therapy is the most effective public health intervention to reduce TB incidence. Within TB preventive treatments, isoniazid, rifampicin, and isoniazid plus rifamycin have effectively reduced the incidence of TB, with isoniazid plus streptomycin combination therapy being the most effective TB preventive treatment option.

**Implications of all the available evidence**

In our study, preventive therapy was found to be the most effective intervention to reduce the occurrence of TB. Therefore, expansion of the provision of TB preventive treatments, through household-level TB screening integrated with the health system would be helpful to reach end-TB strategy milestones and targets. Although WHO has focussed on isoniazid monotherapy and short-time rifamycin-based regimens, our study finds that isoniazid with streptomycin combination is the most likely effective treatment option to reduce the incidence of TB among the population at risk of developing TB. However, only a single old trial has evaluated the efficacy of isoniazid plus streptomycin regimen and the result should be interpreted cautiously. More trials evaluating the efficacy of this regimen in the context of modern TB preventive treatment algorithm are therefore needed.

## Introduction

Other than during the period of some pandemics (such as the recent COVID-19 pandemic), tuberculosis (TB) is the leading infectious cause of death globally, killing more than one million people every year.^1^ It is estimated that more than one-quarter of the world's population is infected with *Mycobacterium tuberculosis* (i.e., latent TB infection), and most cases of active TB arise in people with latent TB infection.^2^ In 2021, there were an estimated 10.6 million incident TB cases and 1.6 million TB-related deaths.^3^ The highest numbers of TB cases were reported in Africa and South-East Asia regions and the vast majority of TB (87%) occurred in 30 high TB-burden countries.^4^ The World Health Organization (WHO) developed the *End-TB Strategy*, which outlines the global approach to prevent and control TB^5^ and is aligned with the Sustainable Development Goals (SDGs).^6^ The strategy has a target of a 90% reduction in TB incidence, to be achieved by the year 2035 relative to 2015 rates.^5^ To attain this ambitious target, it is important to identify the most effective interventions to prevent the development of active TB.

Currently, a range of interventions are available, including TB screening, nutritional therapy, contact investigation, passive case detection, TB preventive therapy, treatment of active TB, and vaccination of children with the Bacillus Calmette–Guérin (BCG) vaccine.^7^ These interventions have various levels of effectiveness with respect to preventing TB incidence in the community. For instance, in a pairwise meta-analysis, it was reported that BCG has shown a 50% protective effect^8^ whereas isoniazid preventive therapy reduced the risk of TB incidence by 74%.^9^ Similarly, existing evidence indicates that screening and treating the latent infection with recommended regimens significantly reduces the progression of latent TB infection to active TB.^10^ However, how these intervention methods compare in terms of reducing the incidence of TB is not well defined, but they can be evaluated using network meta-analysis (NMA).

NMA extends the principles of meta-analysis to evaluate multiple interventions simultaneously in a single analysis.^11^ One of the advantages of NMA is that it allows a quantitative comparison of several interventions that have not been directly compared in primary studies. The other advantage is that it can be used to simultaneously estimate the relative effectiveness of interventions in the evidence network and provide a rank for interventions based on their effectiveness. In contrast, NMA might be affected by heterogeneity since it requires the included studies to be very similar.^12^

Network meta-analysis has been conducted previously for various aspects of TB including treatment of latent TB infection,^13^, ^14^, ^15^ TB prevention among HIV patients,^16^ treatment of drug-resistant TB^17^, ^18^, ^19^ and completion and safety of TB preventive treatments.^20^ However, there is a paucity of evidence on the comparative effectiveness of TB preventive interventions.^21^ Understanding the most effective public health interventions is crucial to design the most effective and efficient solutions whilst considering local resources and capacity constraints. Thus, this study aimed to identify the most effective preventive interventions to reduce the incidence of TB.

## Methods

### Study design

For this study, a systematic review and network meta-analysis of interventional studies was conducted. The study was designed and reported according to the Preferred Reporting Items for Systematic Reviews and Meta-Analysis (PRISMA) extension statement for network meta-analysis.^22^

### Search strategy

We conducted an electronic medical literature search on PubMed, SCOPUS, Web of Science, Cochrane Central Register of Controlled Trials, and ClinicalTrials.gov for relevant trials describing interventions to reduce the incidence of TB, published from the date of each database inception to April 30, 2021. The last search was updated to February 22, 2023, to identify studies published since our initial search. The complete search strategies for all databases are provided in the supplementary material (Supplementary Table S1). Briefly, the search strategy combined terms for tuberculosis AND incidence AND (intervention OR prevention OR control OR random∗ OR “double-blind”) and filters to human studies applied wherever possible. Searches were not restricted by country or date of publication. Reference lists of included papers were screened for additional studies. Corresponding authors were contacted by email in instances where additional information was required, and the Curtin University faculty librarian was consulted for publications where the full text could not be retrieved.

### Study selection and eligibility criteria

All articles identified in the databases were imported to an EndNote library using Endnote version 7. After removing duplicates, the articles were exported to Rayyan^23^ for screening. Three investigators (KAA, BG, and AML) independently screened the titles and abstracts of studies and reviewed full-text articles for inclusion and any differences were resolved through discussion with a fourth reviewer. The following predefined eligibility criteria were used to shortlist studies for analysis: all types of interventional studies that evaluated one or more interventions intended to reduce TB incidence in the community or specific settings (e.g., prison, hospital, or refuge camps) and reported the number of new TB cases diagnosed through sputum microscopy, culture or Genexpert, after each intervention was implemented amongst a population at risk over time. Studies were included if they were randomized controlled trials; quasi-experimental studies, non-randomized trials, with at least two intervention and two control sites; controlled before-after studies with outcome measures before and after the intervention from at least two intervention and two comparable control sites; interrupted time series with a clearly defined point in time for the intervention and outcome measures from at least three-time points in both baseline and intervention periods; or crossover trials. Potential comparators were other prevention interventions, no intervention, or a placebo. We excluded non-interventional studies, conference and meeting abstracts, non-English language articles, animal studies and those that had insufficient information on the main outcome of interest.

### Data extraction

Data were extracted from eligible studies using a predesigned form by the same three investigators. The following data were extracted: study characteristics (first author, year of publication, country of study, study design, settings), participant characteristics (sample size, mean age, age range, sex percentage, inclusion and exclusion criteria of the participants, HIV status, and nonresponse rate), interventions (type, duration, number of participants in intervention and control groups, adherence), and outcomes (number of new TB cases in the intervention and control groups, length of follow-up, person-months at risk in the intervention and control groups, TB diagnostic method). Where studies used the same database, data were extracted only from the most recent and complete reports.

### Quality assessment

Two authors (BG and AML) independently assessed the risk of bias using the revised Cochrane Risk of Bias tool.^24^ The methodology assessed each trial with the following five domains: bias arising from (I) the randomization process; (II) deviations from interventions; (III) missing the outcome data; (IV) measurement of outcome; and (V) selection of the reported result. Each domain had specific signaling questions and responses to these questions led to the judgment of “low risk of bias”, “some concerns” and “high risk of bias”. Therefore, a study with a low risk of bias for all domains and some concern for at least one domain was judged to have “low risk of bias” and “some concerns” respectively. A study with a high risk of bias for at least one domain or that was judged as having some concerns in multiple domains in a way that substantially affected the results was judged to have a “high risk of bias.”

### Statistical analysis

Our primary outcome of interest was the incidence of TB where the number of post-intervention cases were compared with cases in respective control groups. First, the conventional meta-analysis was conducted for studies that directly compared different intervention arms. Then a random-effects Bayesian NMA was performed for direct and indirect comparisons of different interventions. Finally, rank probability and surface area under the cumulative rank curve (SUCRA value) were used to determine intervention rankings. The SUCRA value represents the probability that an intervention is among the best options.^25^ A SUCRA value of 100% indicates the intervention is certain to be the most effective in the network, while a value of 0% indicates it is certain to be the least effective. The larger the SUCRA value, the better the rank of an intervention is in the network.

### Heterogeneity

The conventional pairwise meta-analysis was undertaken using a random-effects model. Pooled RRs with 95% CIs were calculated for direct comparisons of interventions using STATA (version 17.0). Heterogeneity between studies was assessed visually by forest plots and quantitatively by the index of heterogeneity squared (I^2^) statistics, with 95% CI. The I^2^ statistic measures the proportion of observed variance between trials that is not due to chance (rather due to real differences across study populations and interventions). An I^2^ value greater than 75% was interpreted as evidence of substantial levels of heterogeneity. The risk of publication bias was assessed by examining comparison-based funnel plot symmetry and by conducting Egger's regression test where a significant p-value (<0.05) indicates publication bias.

### Network meta-analysis

A random-effects NMA was carried out to summarize direct and indirect (i.e., mixed) evidence. The comparative effectiveness of different interventions such as targeted screening, preventive therapy, nutritional therapy, TB candidate vaccines (Supplementary Table S3), and BCG vaccination) in reducing TB incidence rate was measured using the automated generalized pairwise modeling (GPM) framework. We used the ‘Gemtc’ package for R (Gemtc version 1, Repository CRAN). The pooled estimates were obtained using the Markov-Chain Monte Carlo set on four parallel chains, with 10,000 burn-in iterations, and 200,000 actual simulation iterations. Default prior specification was employed. To check for model convergence, Gelman and Rubin diagnostic plots,^26^ and density plots were used. The network meta-analysis was carried out with R (version 4.2.1, R Foundation for Statistical Computing, Vienna, Austria) and further analysis with STATA (version 17.0, Stata Corp LLC, College Station, TX). In addition, the effectiveness of preventive treatment regimens used in preventive therapy were evaluated through subgroup analysis.

### Network meta-regression, inconsistency, and sub-group analysis

Network meta-regression was used to explore whether the bias has caused heterogeneity in preventive intervention effects. Consistency was explored by examining whether indirect treatment effects (i.e., those that were not directly compared within studies) were similar or different from direct treatment effects (i.e., those that were directly compared within studies) using the node split method. Sensitivity analysis was also conducted by excluding studies with high or some concern of bias based on the judgments obtained from the revised Cochrane risk of bias assessment. A subgroup analysis was also conducted to assess the effectiveness of preventive treatments in people living with HIV/AIDS.

### Ethics

Since this study was a network meta-analysis of data from published literature, an ethical review was not applicable.

### Role of the funding source

The funders of this study had no role in the study design, data collection, data analysis, data interpretation, writing of the report and decision to submit for publication. All authors had full access to all the data in the study and had final responsibility for the decision to submit for publication.

## Results

**Fig. 1:**
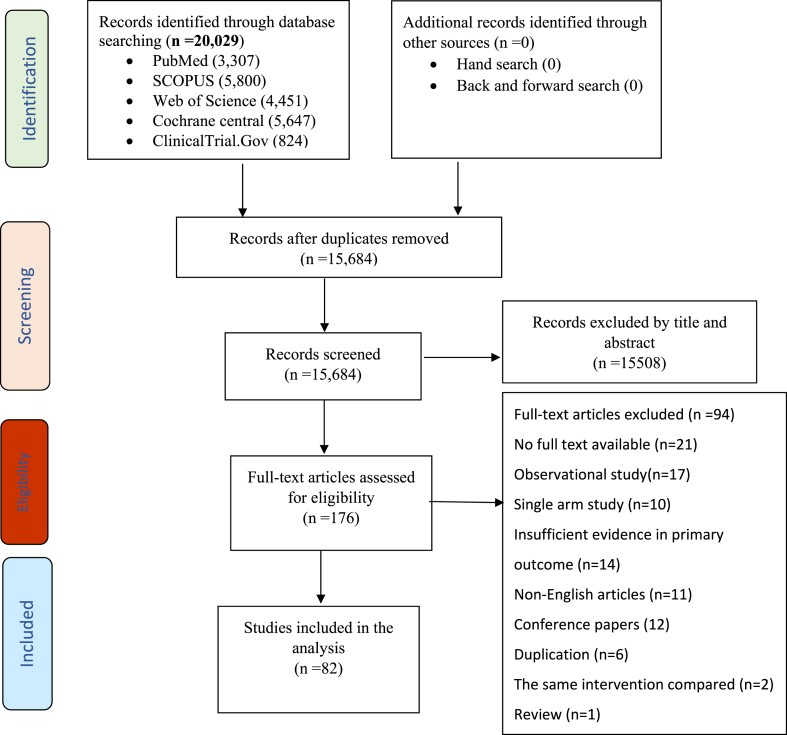
**Summary of systematic review study selection process**.

Through a comprehensive systematic search of different databases, 15,684 studies were identified during the title and abstract screening. After the initial screening, 176 articles were retrieved for full-text assessment and 82 articles were included in this study (Fig. 1).

**Fig. 2:**
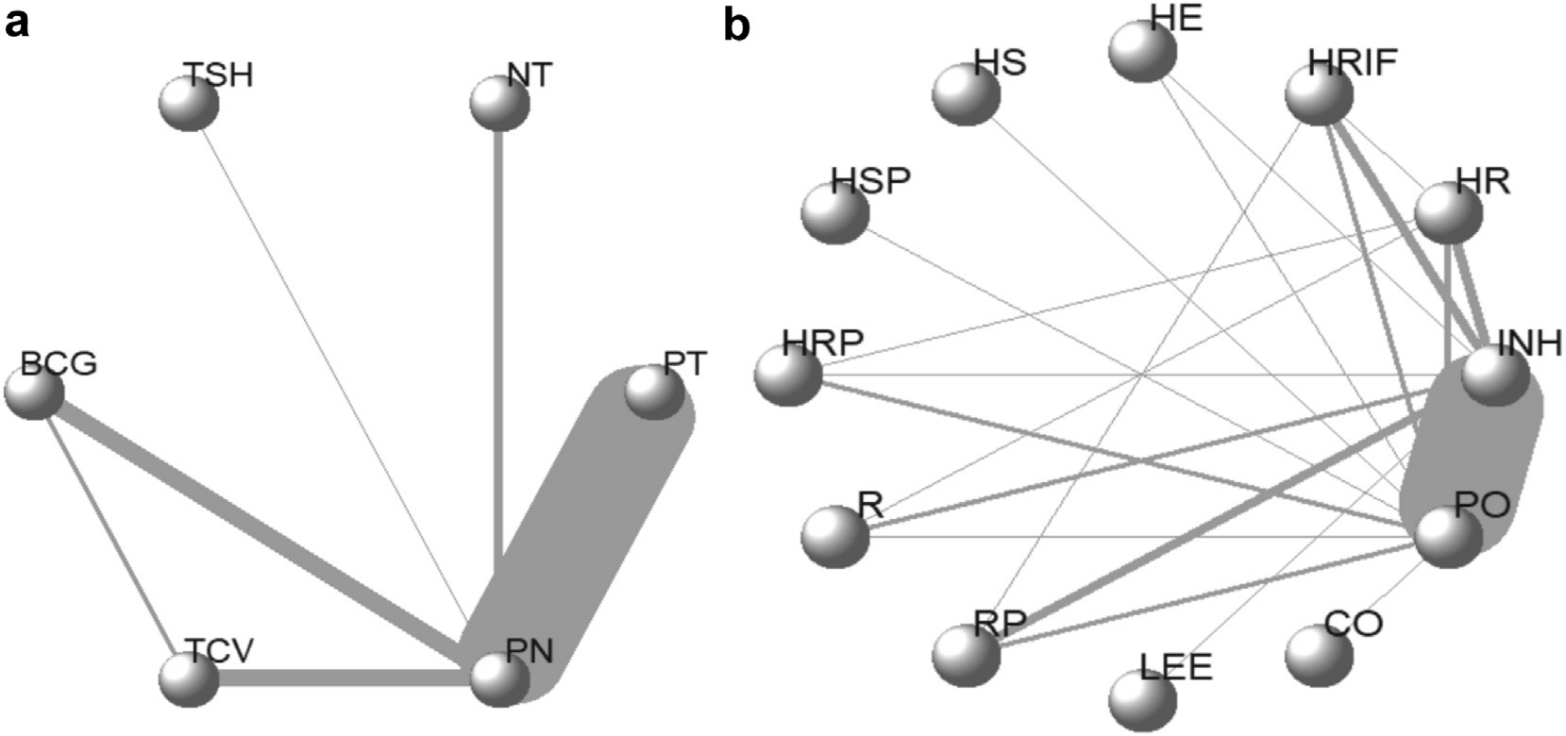
**Network plots for NMA of preventive public health interventions (a) and preventive treatments (b)**. Lines connect interventions or treatments that have been investigated in head-to-head (direct) comparisons in the included studies. The width of the lines indicates the cumulative number of studies for each direct comparison. Different nodes refer to different public health interventions (a) and preventive treatments (b). Accordingly in Fig. 2a, acronyms “PT”, “NT”, “TBH”, “BCG”, “TCV” and “PN” refer to preventive therapy, nutritional therapy, targeted screening plus isoniazid preventive therapy, Bacillus Calmette-Guérin vaccination, TB candidate vaccines and placebo or no intervention respectively; whereas in Fig. 2b, “INH”, “HR”, “HRIF”, “HE”,“HS”, “HSP”,“HRP”, “R”, “RP”, “LEE”, “CO” and “PO” refer to isoniazid, isoniazid plus rifampicin, isoniazid plus rifapentine, isoniazid plus ethambutol, isoniazid plus streptomycin, isoniazid plus sulphadoxine pyrimethamine, isoniazid plus rifampicin plus pyrazinamide, rifampicin, rifampicin plus pyrazinamide, levofloxacin, cotrimoxazole and placebo or no treatment respectively.

In the NMA of preventive interventions, some studies that used active intervention as a control group and, therefore, could not be connected to the network of direct evidence in the network plot, were excluded (n = 13). Therefore, 69 studies were included in the NMA of preventive interventions. However, these 13 excluded studies were included in a subgroup analysis, which allowed a multi-arm network geometry (Fig. 2b) in the NMA of preventive treatments (n = 62).

### Characteristics of the included studies

A total of 69 interventional studies which reported five active TB preventive public health interventions including TB preventive therapy, nutritional therapy, targeted TB screening plus isoniazid preventive therapy (IPT), BCG vaccination, and vaccination with new TB candidate vaccines were included in the Bayesian network meta-analysis of preventive interventions (Fig. 2a). All studies used no intervention or placebo as a reference intervention.

Overall, 1,144,520 participants were included in the NMA of preventive interventions. Of these, 55,637 (4.86%) participants were specifically assigned to specific TB preventive therapies including isoniazid, rifampicin, pyrazinamide, rifapentine, streptomycin, cotrimoxazole, levofloxacin or combination of any of these treatments. In addition, 81,279 (7.01%) participants were allocated to nutritional therapies such as multivitamins, selenium, and vitamin D3 supplementation. A total of 483,039 (41.50%) participants were assigned to BCG vaccination, 21,717 (1.9%) to TB candidate vaccines, and 10,752 (1.01%) to targeted TB screening plus isoniazid preventive treatment. The population preventive fraction for each intervention was computed and the finding indicates that 33.64%, 19.78% and 14% of all TB cases that would have otherwise developed in the population have been prevented by preventive therapy, TB candidate vaccines and BCG vaccination respectively (Supplementary Table S2).

For the NMA of the effectiveness of preventive treatments, a total of 62 articles that compared the efficacy of preventive treatments in the head-to-head analysis were included. Three publications included two studies, giving a total of 65 studies. In these interventional studies, 11 active treatment regimens, including isoniazid, isoniazid plus rifampicin, isoniazid plus rifapentine, isoniazid plus ethambutol, isoniazid plus streptomycin, isoniazid plus sulphadoxine-pyrimethamine, isoniazid plus rifampicin plus pyrazinamide, rifampicin, rifampicin plus pyrazinamide, levofloxacin, and cotrimoxazole were compared (Fig. 2b).

The majority of included studies exhibited a low risk of bias although some studies were judged to have a high risk of bias.^27^, ^28^, ^29^, ^30^, ^31^, ^32^, ^33^, ^34^, ^35^ The characteristics of 82 included studies along with the risk of bias assessment is provided in the supplementary file (Supplementary Fig. S1).

Although the asymmetrical presentation of the comparison-adjusted funnel plot indicates potential publication bias (Supplementary Fig. S3), it was not statistically significant when investigated by Egger's regression test (p-value = 0.99). Similarly, no statistically significant evidence of inconsistency was reported in the node splitting test for both NMA of preventive interventions (Supplementary Fig. S4) and that of preventive treatments (Supplementary Fig. S7). In the pairwise meta-analysis of the preventive interventions, no statistical evidence of heterogeneity was observed for most comparisons except in one comparison (BCG vs placebo or no intervention) where high heterogeneity (I^2^ = 93.17%) was recorded. Overall, moderate heterogeneity (I^2^ = 39.00%) was observed, indicating reasonable variation between studies included in this NMA (Supplementary Fig. S2).

### Network meta-analysis results

**Fig. 3:**
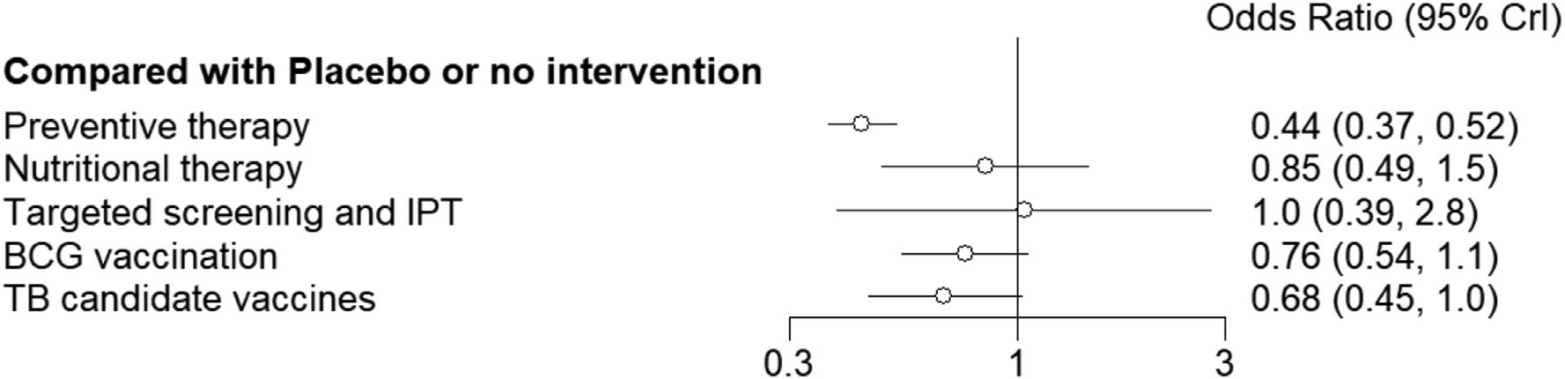
**Forest plot of relative effects of TB preventive interventions**. Each horizontal line on forest plot represents the pooled Odds ratio of individual interventions (compared with placebo/none), with the odds ratio plotted as a circle and the 95% credible interval plotted as the line. When the odds ratio is less than 1, the specified intervention is associated with lower risk of TB incidence.

**Fig. 7:**
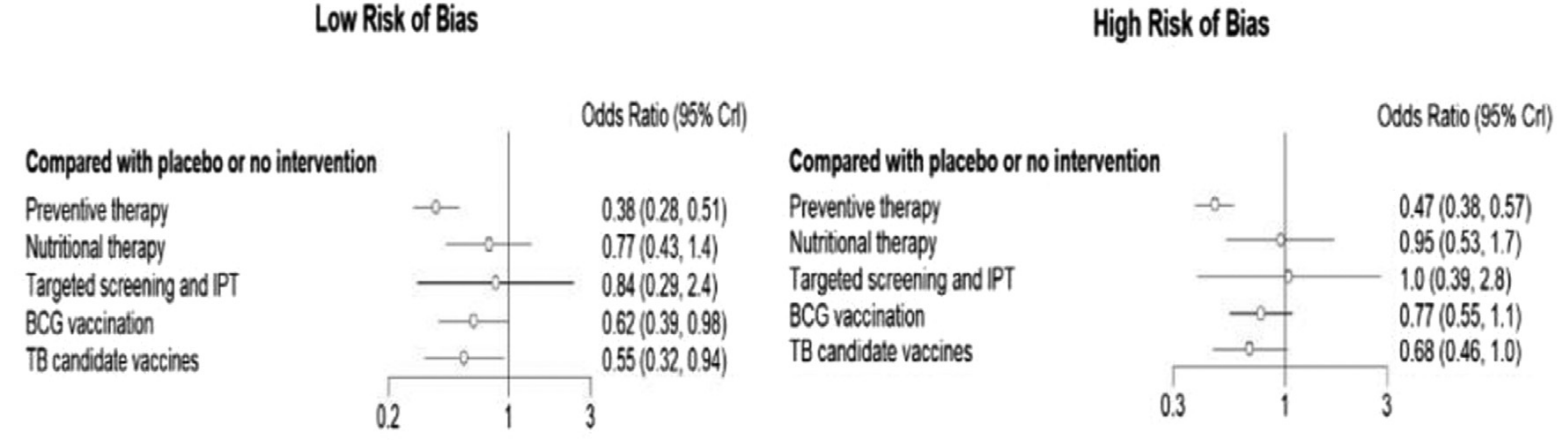
**Comparison based forest plots of network meta regression of preventive interventions**.

In the network meta-analysis of preventive interventions, five active interventions have been compared. Fig. 3 presents the relative effectiveness of all possible pairs of interventions with their level of uncertainty. When all interventions were considered, TB preventive therapy significantly reduced the incidence of TB with the highest point estimate (OR = 0.44, 95% CrI 0.36–0.52). Although other interventions have clinical significance with respect to reducing TB incidence, they did not reach statistical significance in this analysis (Fig. 3). However, BCG vaccination (OR = 0.62, 95% CrI 0.39–0.98) (Fig. 7) and TB candidate vaccines (OR = 0.67, 95% CrI 0.46–0.98) (Supplementary Fig. S5) significantly reduced the incidence of TB as shown in the network meta-regression and sensitivity analysis respectively.

**Fig. 4:**
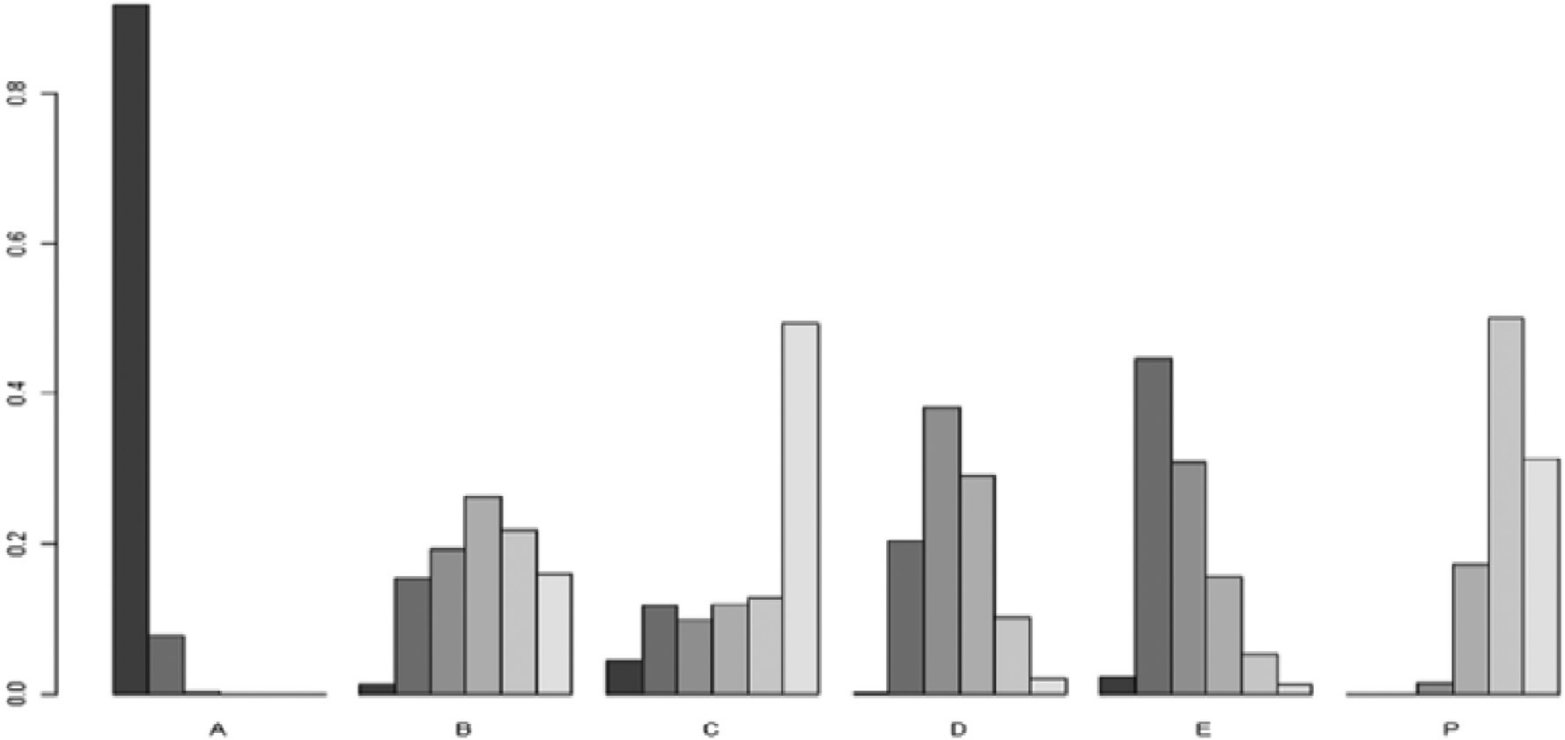
**Rank of preventive interventions with respect to reducing TB incidence**. The Y-axis indicates the probability of being the first the second and so on most likely effective intervention. The X-axis indicates the specific intervention that has been compared. Thus, “A”, “B”, “C”, “D”, “E” and “P” refer to preventive therapy, nutritional therapy, screening plus IPT, BCG vaccination, TB candidate vaccines and placebo or no intervention respectively.

Moreover, based on the results of the Bayesian NMA, TB preventive therapy was ranked as the best intervention for reducing TB incidence as compared to all available TB preventive public health interventions, followed by vaccination (Fig. 4).

Ranking interventions based on rank probability has some limitations since it doesn't account for the uncertainty between relative effect estimates and relative ranking. Due to this limitation, the SUCRA value which uses mean or cumulative ranking probabilities^36^ has been applied to evaluate rankings with the acknowledgment that this metric considers the effect estimate and does not consider the precision.^37^ Thus, the SUCRA values for each preventive intervention were detailed below and preventive therapy was ranked as the best intervention (Table 1).

**Table 1:** Surface area under the cumulative ranking probability curve values for each intervention.

Interventions	SUCRA value (%)	Rank
Preventive therapy	98.28	Best
TB candidate vaccines	63.90	Second
BCG vaccination	53.04	Third
Nutritional therapy	40.05	Fourth
Targeted screening and IPT	26.94	Fifth
Placebo or no intervention	17.75	Last

**Fig. 5:**
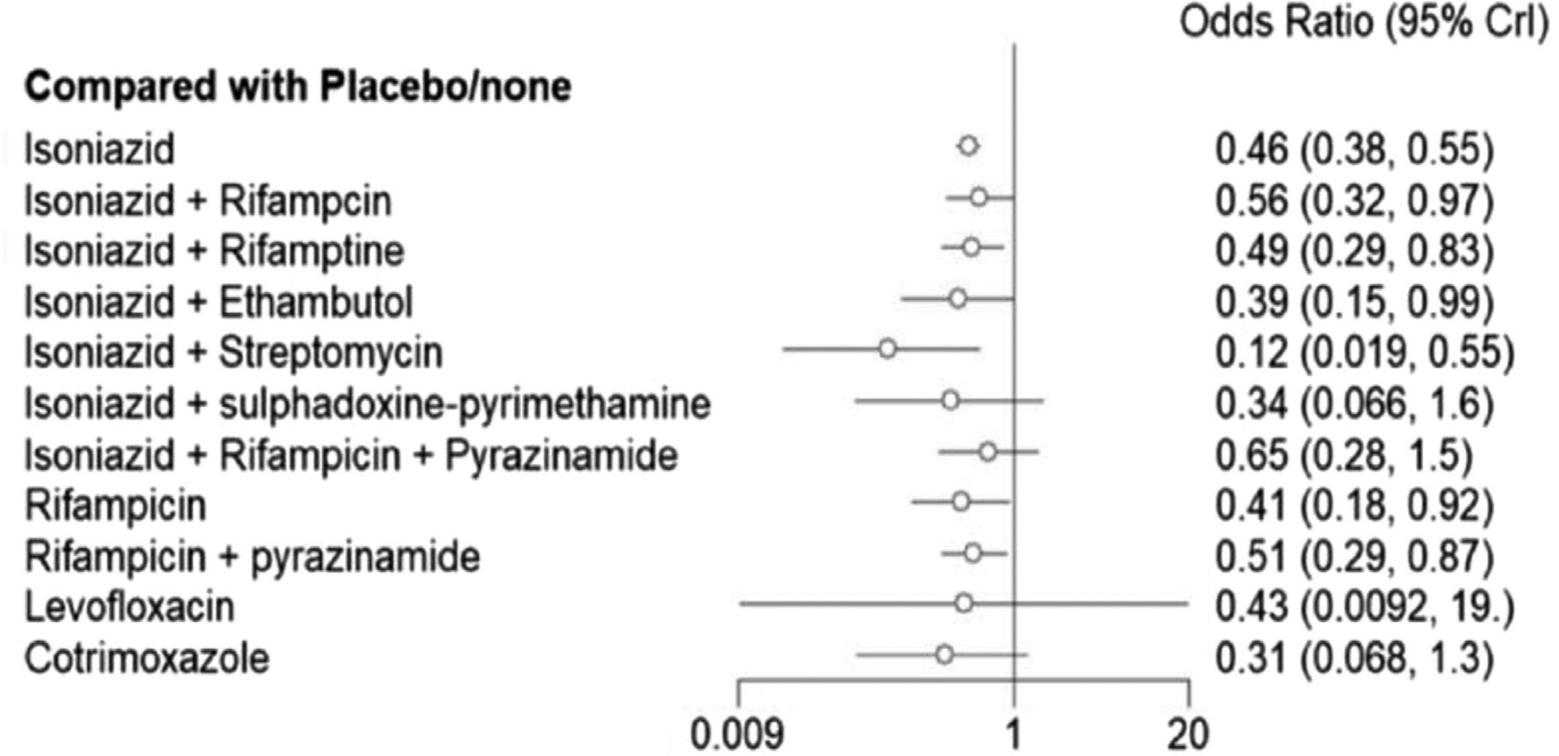
**A forest plot of relative effects of preventive treatments compared to placebo or no treatment**.

In the sub-group analysis of preventive treatments, we found that seven treatment options, including isoniazid (OR = 0.46, 95% CrI 0.38–0.55), isoniazid plus rifampicin (OR = 0.56, 95% CrI 0.32–0.97), isoniazid plus rifapentine (OR = 0.50, 95% CrI 0.29–0.83), isoniazid plus ethambutol (OR = 0.39, 95% CrI 0.15–0.99), isoniazid plus streptomycin (OR = 0.12, 95% CrI 0.02–0.56), rifampicin (OR = 0.41, 95% CrI 0.18–0.91), and rifampicin plus pyrazinamide (OR = 0.51, 95% CrI 0.29–0.87) were significantly more effective than placebo or no intervention in reducing TB incidence (Fig. 5).

**Fig. 6:**
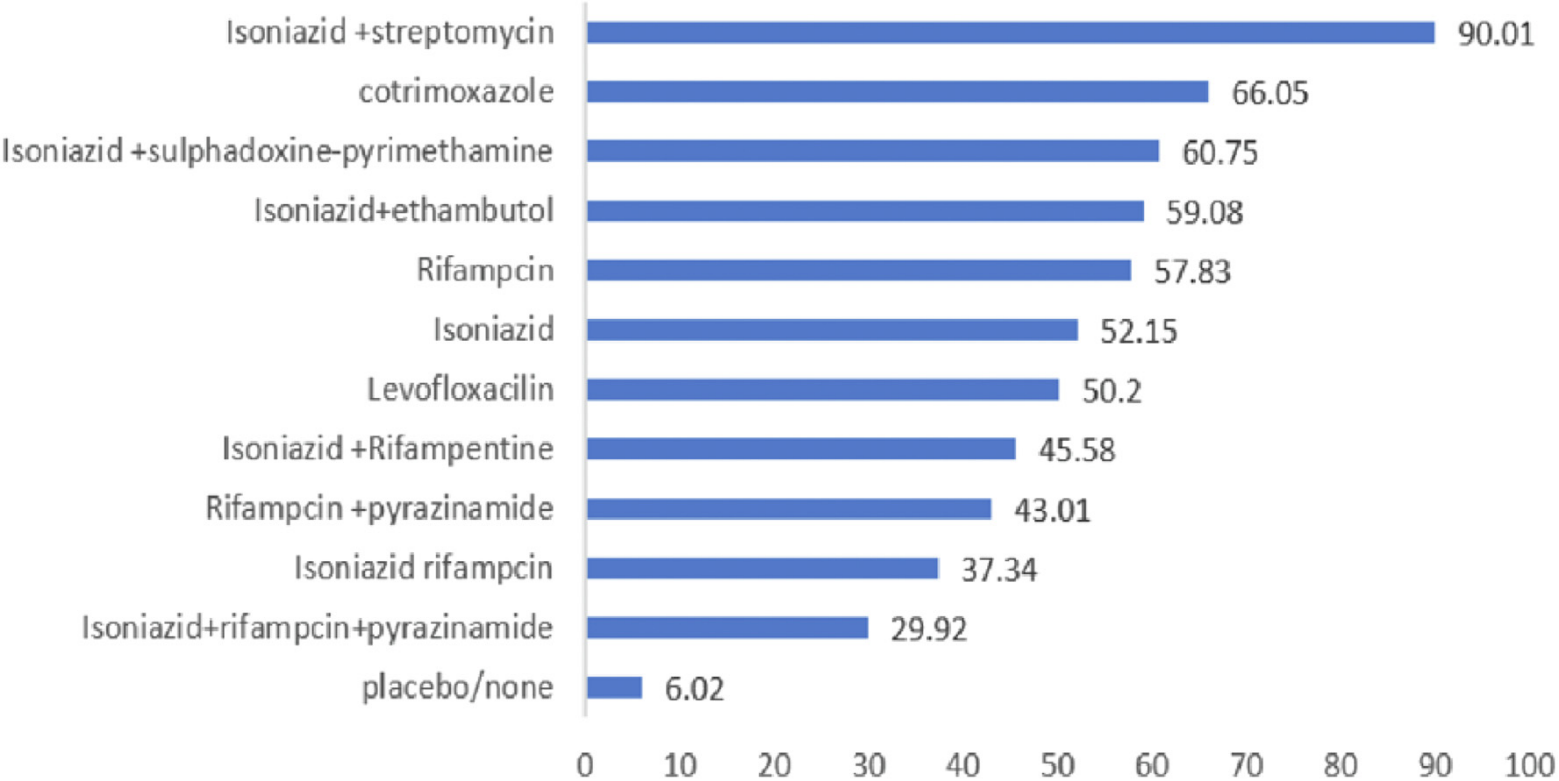
**Surface area under cumulative rank probability curve (SUCRA) values of preventive treatments**.

We have further investigated the comparative effectiveness of TB preventive treatments using rank probability and SURA values. The findings consistently indicate that isoniazid and streptomycin combination therapy to be the most effective treatment for reducing TB incidence (Fig. 6 and Supplementary Fig. S8).

### Network meta-regression and sensitivity analysis results

This study has also investigated the effect of bias on the findings of preventive interventions through network meta-regression by using risk of bias as a covariate and the findings indicate that bias did not significantly influence the reported results (β = −0.21, 95% CrI −0.57, 0.13). This was again supported by the deviance information criteria (DIC) value, which favoured the normal NMA to have the best fit to data (Supplementary Table S3). We have also explored the effect of bias by generating two separate forest plots of relative effects i.e., one for a high risk of bias and the other when it is low. Comparing the forest plots, the relative effects have overlapping confidence intervals, which indicates the absence of bias. However, a difference in the actual point estimate and level of significance was observed for some interventions including BCG vaccination (Fig. 7).

For NMA of preventive interventions, a sensitivity analysis was also conducted by excluding high-risk and some concern of risk studies to detect the source of heterogeneity and to assess the effect of bias. Accordingly, we found consistent intervention ranks (Supplementary Fig. S6) though there has been a difference in the significance level of relative effect estimates for a few interventions including TB candidate vaccines (Supplementary Fig. S5).

## Discussion

In this Bayesian NMA, we reviewed the comparative effectiveness of various public health interventions including TB preventive treatment, nutritional therapy, TB screening and IPT, BCG vaccination, and TB candidate vaccines in reducing TB incidence. We found that TB preventive therapy was the most effective intervention in reducing the incidence of TB among the population at risk of developing active TB.

TB preventive therapy is a treatment offered to individuals to prevent initial infection^38^ and the progression of latent infection to clinical disease. The World Health Organization (WHO) recommends focussing on providing preventive treatment to high-risk populations such as people living with HIV and household child contacts under 5 years of age. In 2018, it recommended extending the focus to all household contacts of bacteriologically confirmed pulmonary TB cases and other high-risk groups including recipients of dialysis, patients in preparation for solid organ or a hematologic transplant, the homeless, prisoners and people with silicosis.^39^

Despite its proven effectiveness, TB preventive therapy is globally highly underutilized. For instance, although ambitious preventive treatment targets were enacted by the first United Nations high-level meeting on TB in 2018, only 8.7 million people were provided preventive treatment in a three-year (2018–2020) period which is only 29% of the 5-year target.^40^ This indicates that substantial expansion efforts, including household-level TB screening integrated with the health system, are needed to improve the provision of TB preventive treatments to reach end-TB strategy milestones and targets.

We have also investigated the effectiveness of preventive treatment options for reducing TB incidence among the population at risk of developing TB. A total of 11 active treatment regimens were directly compared in 62 trials and we found isoniazid, one of the drugs recommended for preventive treatment in the current WHO guideline, is superior to placebo or no treatment in reducing TB incidence. Besides, in the subgroup network meta-analysis isoniazid significantly reduced the incidence of TB among HIV-positive patients (Supplementary Fig. S9). Consistently, the efficacy of isoniazid in HIV-infected patients has been documented in previous pair-wise meta-analyses.^41^ The existing evidence also indicates that isoniazid preventive therapy is safe in individuals being treated with anti-retroviral therapy.^42^ Despite emerging evidence of toxicity risk and lower treatment completion rates than shorter rifamycin-based regimens, isoniazid monotherapy is also reported to be efficacious to treat LTBI.^43^

We also found that rifamycin-based regimens such as a combination of isoniazid with either rifampicin or rifapentine and rifampicin alone significantly reduced TB incidence. Although rifamycin-based regimens have proven drug interactions due to their cytokine-inducing effect, WHO recommends access to these short-term regimens to improve treatment completion rates, and they have currently been endorsed by 36 countries worldwide.^39^

Of all investigated preventive treatment options, the isoniazid plus streptomycin regimen was ranked to be the most likely best treatment option to reduce TB incidence among populations at risk of developing TB. However, this preventive treatment regimen is not included in the current WHO consolidated TB preventive treatment guidelines^39^ which might be partly due to the route of administration hindrance since streptomycin has to be administered by regular intramuscular injections^44^ which might lead to poor adherence. However, this result should be interpreted with caution as it is based on a single old trial that investigated isoniazid plus streptomycin efficacy in preventing TB incidence amongst the study population with residual cavitation but no active TB. Besides, because of the lack of indirect loops involving this treatment option, the node splitting test could not evaluate the inconsistency assumption for the comparative efficacy of this regimen. Therefore, further trials are needed to verify its efficacy in participants which are typically included in the modern TB preventive treatment (TPT) strategy to consider as a potential treatment option in future guidelines.

Our study has several strengths. It is the first comprehensive Bayesian NMA, that compared preventive public health interventions and treatment regimens for preventing active TB that have not been compared in either head-to-head studies or in conventional meta-analysis. Second, we reported our findings according to the PRISMA-NMA statement and did not find statistical evidence of inconsistency and publication bias. Finally, although an overall substantial heterogeneity was recorded, no heterogeneity was observed in most direct comparisons of preventive interventions.

However, like that of pairwise conventional meta-analysis, the validity of NMA relies on the homogeneity of included studies^45^ and the observed heterogeneity might have affected the reported findings. Besides, data abstracted for NMA should meet consistency and transitivity assumptions for drawing valid conclusions.^11^ For NMA of preventive interventions, inconsistency was statistically evaluated only for one comparison (BCG vs TB candidate vaccines) as other interventions were not indirectly connected in the network loop. Although no statistical evidence of inconsistency was observed, it cannot be fully excluded as it was assessed only for a single comparison. Moreover, the consistency assumption is valid when all included studies are jointly randomizable, which requires the study participants in a particular study to be eligible for inclusion in the other studies^46^ which is not the case in the current study.

On the other hand, inconsistency was tested for multiple comparisons for preventive treatment regimens and the node splitting test returns statistically insignificant findings that indicate the absence of inconsistency. However, it was still infeasible to test for all comparisons and the 95% CrI for some estimates that were returned in the node splitting test was wide and inconsistency could not be fully excluded in our study.

Another important assumption in NMA is transitivity which requires the similarity of population groups and methods employed in the primary studies. In this regard, a large difference in the target populations in intervention trials for both NMAs (preventive interventions and preventive treatments) could make the distribution of effect modifiers including TB infection, and re-infection uneven. This might have influenced the likelihood of transitivity which may in turn affect the validity of findings.

Another limitation of our study was that we only evaluated the treatment effects of the major classes of interventions since it was not feasible to form a connected network for a more refined classification of interventions with the same follow-up periods and doses of treatments due to insufficient study participants and the number of events. Thus, the difference in the length of follow-up and variations in the dose of the treatment could contribute to variations in the study outcomes. In addition, articles that were excluded e.g., due to the absence of full text might have influenced the current results. Finally, to adjust the effect of potential confounders including the burden of TB in the countries of the included articles, sociodemographic and economic factors, a subgroup analysis should ideally be performed. However, the study size assigned to each comparison group was too small and getting connected networks was then not feasible.

Our study found that TB preventive therapy was the most likely effective public health intervention in reducing TB incidence. Within TB preventive treatments, isoniazid, rifampicin, and isoniazid plus rifamycin have effectively reduced the incidence of TB, with isoniazid plus streptomycin combination therapy having the highest probability of being the most effective TB preventive treatment option. This comparative information could potentially be an input for policymakers and stakeholders as they need to see the relative effectiveness of different preventive strategies whilst considering local resources and capacity.

## Contributors

KAA, ACAC and BG designed the study. AML and BG conducted data curation. AML and KAA conducted statistical analysis and drafted the manuscript. KAA, ACAC and BG performed critical review and editing. All authors have full access to data and approved the submission.

## Data sharing statement

Data will be available upon request from the corresponding author.

## Declaration of interests

All authors declare that they have no competing interests.

## Appendix A Supplementary data

Suplementary file
