# Supplementary material for: Comparative effectiveness of interventions for preventing tuberculosis: systematic review and network meta-analysis of interventional studies
Source: eClinicalMedicine. 2023 Sep 9;64:102209. doi: 10.1016/j.eclinm.2023.102209 (PMC10507197; doi:10.1016/j.eclinm.2023.102209)

Contents

Supplementary Tables **2**

Suplementary Table 1: Search strategy 2

Supplementary Table 2: Population preventive fraction for each intervention. 3

Supplementary Table 3: Model comparison and coefficient of risk of bias. 3

Supplementary Table 4: Description of 82 studies included in the network meta-analysis. 4

Suplementary Figures **8**

Suplementary Figure 1: Quality of included trials 8

Supplementary Figure 2: Pair wise meta-analysis of preventive interventions. 9

Supplementary Figure 3: Comparison based funnel plot of preventive interventions of TB incidence. 10

Supplementary Figure 4: Node splitting inconsistency test for preventive interventions. 10

Supplementary Figure 5: Forest plot of relative effects for sensitivity analysis of preventive interventions excluding high risk and some concern studies. 11

Supplementary Figure 6: Ranking probabilities of preventive interventions of TB excluding high risk and some concern studies. 11

Supplementary Figure 7: Node splitting inconsistency test for efficacy of preventive treatments. 12

Supplementary Figure 8: Ranking probabilities of effectiveness of preventive treatments of TB. 13

Supplementary Figure 9: Subgroup analysis of effective preventive treatments among PLHIV 13

SUPPLEMENTARY TABLES

Suplementary Table 1: Search strategy

Search	Query	Results
**PubMed**		
#1	tuberculosis [MeSH] OR mycobacterium tuberculosis [MeSH] OR tuberculosis"[Title/Abstract] OR tuberculosis [Title/Abstract] OR TB[Title/Abstract]	300,032
#2	(Clinical trial OR Randomized Controlled Trial OR controlled trial OR cross over study OR cross over trial OR single blind OR double blind OR factorial design OR factorial trial OR interventional studies)	7,346,075
#3	#1 and #2	51,667
Limit to	Human and English	**3307**
**SCOPUS**		
#1	("tuberculosis" OR "TB")	384,308
#2	("clinical trials" OR "randomized controlled trial" OR "controlled clinical trial" OR "random allocation" OR "randomly allocated" OR "allocated randomly" OR "Double-Blind Method" OR "Single-Blind Method" OR "Cross-Over Studies" OR "Placebos" OR "cross-over trial" OR "single blind" OR "double blind" OR "factorial design" OR "factorial trial" OR "clinical trial*" OR "trial*" OR "rct" OR "rcts" OR "random*" OR "blind*")	4,508,878
# 3	("epidemiology" OR "incidence" OR "prevalence" OR "risk" OR "ratio" OR "eliminate*" OR "eradicate*" OR "prevent*" OR "control*" OR "intervent*")	23,326,799
# 4	# 1 AND #2 AND #3	14,508
Limit to	Human AND English AND Tuberculosis	**5,800**
**Web of Science**		
#1	(Tuberculosis OR TB)	205,616
#2	("clinical trial* " OR "randomized controlled trial*" OR "random allocation" OR "randomly allocated" OR "allocated randomly" OR "cross over study*" OR "cross over trial" OR "single blind" OR "double blind" OR "factorial design" OR "factorial trial")	937,438
#3	#1 AND #2	4,451
**Cochrane Central Register of Controlled Trials**		
#1	Tuberculosis	7442
#2	TB	6532
#3	#1 OR #2	11570
#4	Epidemiology	63769
#5	Incidence	124754
#6	Prevalence	40599
#7	Risk	246625
#8	Ratio	124213
#9	"eliminate*"	4210
#10	"eradicate*"	1022
#11	"prevent*"	51858
#12	"control*"	491000
#13	"intervent*"	104
#14	#4 OR #5 OR #6 OR #7OR #8 OR #9 OR #10 OR #11 OR #12 OR #13	783849
	#4 AND #14	6149
Limit to	Trials	**5,647**
**ClinicalTrials.gov**		
#1	Tuberculosis	
#2	Interventional studies	
#3	Interventional Studies AND Tuberculosis	**824**

Supplementary Table 2: Population preventive fraction for each intervention.

Interventions	TB disease Yes	TB disease No	Total	RR	Proportion of exposed (P)	Population preventive fraction [P(1-RR) x 100]
Preventive therapy	1338	54299	55637	0.42	0.58	33.64
Control group	2189	38302	40473			
Nutritional therapy	340	80939	81279	1.14	0.53	NA
Control group	266	71015	71281			
Targeted screening + IPT	254	10498	10752	1.04	0.53	NA
Control group	221	9487	9708			
BCG vaccination	1469	481570	483039	0.76	0.58	13.92
Control group	1406	353611	355017			
TB candidate vaccines	204	21513	21717	0.54	0.43	19.78
Control group	498	28291	28789			

Note: RR: relative risk; TB: tuberculosis; NA: not applicable

Supplementary Table 3: Model comparison and coefficient of risk of bias.

parameters	Normal network meta analysis	Network meta regression
Deviance information criteria (DIC	259.3499	259.6917
β (95% CI)	-	-0.21, 95% CrI -0.57- 0.13

Supplementary Table 4: Description of 82 studies included in the network meta-analysis.

Study ID	Year	Country	Study population	Study design	Intervention	Control	follow up (months)	Excluded in preventive interventions	Included in preventive treatments	Included in NMA of PLHIV
Agarwal	2004	India	Renal transplant recipients	RCT	Isoniazid	No intervention	30		✓	
Ahmed	2005	Pakistan	Allogeneic stem cell transplant recipients	Non-randomized quasi experimental	Isoniazid	No intervention	18.6		✓	
Apriani	2022	Indonesia	children and adults with latent TB infection	RCT	Isoniazid	Rifampicin	28	✓	✓	
Barreto	2011	Brazil	BCG vaccinated school aged children	Cluster randomized control trial	BCG revaccination	No intervention	108			
Bunyasi	2017	South Africa	Children with LTBI infection	RCT	Isoniazid	No intervention	60		✓	
Bush	1965	Japan	Household TB contacts	RCT	Isoniazid	placebo	12		✓	
Campa	2017	Botswana	PLHIV on ART	RCT	Multivitamin	Placebo	24			
Campa	2017	Botswana	PLHIV not on ART	RCT	Selenium	Placebo	24			
Campa	2017	Botswana	PLHIV not on ART	RCT	Multivitamin + Selenium	Placebo	24			
Churchyard	2003	South Africa	Miners with HIV	RCT	Isoniazid	No intervention	12		✓	
Churchyard	2014	South Africa	Gold miners without active TB	cluster randomized trial	Isoniazid + pyridoxine	No intervention	12		✓	
Churchyard	2021	SEM	HIV patients with no active TB	RCT	Isoniazid-rifapentine	Isoniazid	24 vs 12	✓	✓	
Comstock	1979	Alaska	Eskimos without active TB	RCT	Isoniazid	Placebo	-		✓	✓
Comstock	1967	Alaska	Community members without active TB	RCT	Isoniazid	No intervention	69.3		✓	
Comstock	1966	Southern US	Non-reactors to TST >5mm	Controlled trial	BCG	No intervention	168			
Comstock	1974	Alaska	Community without active TB	controlled trial	Isoniazid	Placebo	90-180		✓	
Comstock	1976	US	Community without active TB	controlled trial	BCG	No intervention	240			
Cowie	1996	South Africa	Gold miners with chronic silicosis	RCT	Rifampicin isoniazid pyrazinamide	Placebo	48		✓	
Crook	2016	Uganda + Zimbabwe	HIV infected children on ART	RCT	co-trimoxazole	No intervention	24		✓	✓
Debre	1973	France	LTBI	controlled trial	isoniazid	No intervention	120		✓	
Dias de Oliveira	2020	Brazil	QFT reactors	RCT	Isoniazid	Placebo	6		✓	
Dorken	1984	Canada	tuberculin reactors	RCT	isoniazid + ethambutol	No intervention	120		✓	
Durovni	2013	Brazil	HIV positive patients on ART	cluster randomized trial	Targeted screening +IPT	No intervention	30			
Egsmose	1965	Kenya	TB contacts that are tuberculin non-reactors	RCT	Isoniazid	Placebo	12		✓	
Egsmose	1965	Kenya	TB contacts that are tuberculin reactors	RCT	Isoniazid	Placebo	12			
Falk	1978	US	Veterans with inactive TB	RCT	Isoniazid	placebo	60		✓	
Fitzgerald	2000	Haiti	HIV positive	RCT	Isoniazid	placebo	24		✓	✓
Frigati	2011	South Africa	HIV positive children	RCT	Isoniazid	placebo	48		✓	✓

Gao	2018	China	Rural residents with LTBI	RCT	Rifapentine + isoniazid	isoniazid + rifamycin	24	✓	✓	
Golub	2015	Brazil	HIV positive with reactive TST	Cluster randomized trial	Isoniazid	No intervention	56.4		✓	✓
Gordin	2000	UMHB	HIV positive with reactive TST	RCT	Rifampin plus pyrazinamide	Isoniazid	36	✓	✓	✓
Gordin	1997	US	HIV positive with anergy	RCT	Isoniazid	Placebo	33		✓	✓
Gray	2014	South Africa	HIV positive children on ART	RCT	Isoniazid	Placebo	38.5		✓	✓
Grzybowski	1976	Canada	Inactive disease in TB patients who had received inadequate chemotherapy	non-randomized controlled trial	Isoniazid	None or placebo	-		✓	
Haller	1999	Ivory Coast	HIV positive	RCT	Isoniazid + sulphadoxine-pyrimethamine	No intervention	24		✓	✓
Halsey	1998	Haiti	HIV positive PPD reactive	RCT	Rifampicin + pyrazinamide	Isoniazid + pyridoxine	30	✓	✓	✓
Hart	1977	Great Britain	participants with non-reactive skin test	RCT	BCG vaccine	No intervention	240			
Hart	1977	Great Britain	participants with non-reactive skin test	RCT	vole bacillus vaccine	No intervention	240			
Hawken	1997	Kenya	HIV positive adults	RCT	Isoniazid	Placebo	21.96		✓	✓
TB research center	1992	Hong Kong	men silicosis of any severity	RCT	Rifampin,	Placebo	60		✓	
TB research center	1992	Hong Kong	men silicosis of any severity	RCT	Rifampin + Isoniazid	Placebo	60			
TB research center	1992	Hong Kong	men with silicosis of any severity	RCT	Isoniazid	Placebo	60			
Horwitz	1974	Greenland	Tuberculin reactors no history of active TB	RCT	Isoniazid	Placebo	144		✓	
IUAT	1982	CFGHPRY	fibrotic pulmonary lesions of tuberculosis	RCT	Isoniazid	Placebo	60		✓	
Jimenez Fuentes	2013	Spain	Immigrants with LTBI	RCT	Isoniazid	isoniazid + rifampicin	60	✓	✓	
Karonga Prevention Trial	1996	Malawi	individuals lacking BCG scar	RCT	BCG+killed M leprae	BCG	60-108			
Karonga Prevention Trial	1996	Malawi	individuals with BCG scar	RCT	BCG	Placebo	60-108			
Katoch	2008	India	Healthy contacts of leprosy patients	RCT	Mw vaccine (TB candidate vaccine)	Placebo	120-156			
Khawcharoenporn	2012	Thailand	HIV patients	Controlled trial	Isoniazid	No intervention	48		✓	✓
Kim	2015	South Korea	Kidney transplant recipients with reactive IGRA	RCT	isoniazid	No intervention	21.6		✓	
LaCourse	2021	Kenya	HIV exposed uninfected infants	RCT	isoniazid + pyridoxine	No intervention	12		✓	✓
Luabeya	2015	South Africa	HIV negative infants with QFT-GIT reactive	Controlled TB trial	Isoniazid	No intervention	36		✓	
Madhi	2011	South Africa	HIV positive infants exposed to TB case	RCT	Isoniazid	Placebo			✓	✓
Martison	2011	South Africa	HIV positive adults with reactive TB skin test	RCT	rifapentine + isoniazid	Isoniazid	48	✓	✓	✓
Martison	2011	South Africa	HIV positive adults, with TB skin test reactive, b	RCT	Rifampin plus isoniazid	Isoniazid	49.2			

Menzies	2018	9 countries	Adults with positive skin test	RCT	Rifampin	Isoniazid	28	✓	✓	
Mohammed	2007	South Africa	TST negative HIV positive Adults on ART	RCT	isoniazid + pyridoxine	Placebo	12		✓	✓
Munseri	2020	Tanzania	13–15-year-old with negative T-SPOT TB IGRA	RCT	DAR-901 (TB candidate vaccine)	Placebo	36			
Mwinga	1998	Zambia	> 15 years old with HIV	RCT	rifampicin + pyrazinamide	Placebo	21.6		✓	✓
Mwinga	1998	Zambia	> 15 years old with HIV	RCT	Isoniazid	Placebo	21.6			
Naqvi	2006	Pakistan	Renal allograft recipients	RCT	isoniazid + pyridoxine	No intervention	12		✓	
Naqvi	2010	Pakistan	Renal transplant recipients	RCT	Isoniazid	No intervention	48		✓	
Nazareth	1971	India	persons with residual cavitation but no active TB	RCT	isoniazid + pyridoxine	Placebo	48		✓	
Nazareth	1971	India	Persons without residual cavitation no active TB	RCT	Isoniazid	Placebo	48			
Nazareth	1977	India	persons with residual cavitation but no active TB	RCT	Streptomycin + isoniazid	Placebo	48		✓	
Ndiaye	2015	South Africa + Senegal	HIV positive adults	RCT	MVA85A vaccine (TB candidate vaccine)	Placebo	6			
Nemes	2018A	South Africa	Adolescents With negative QFT	RCT	H4:IC31 (TB candidate vaccine)	Placebo	24			
Nemes	2018A	South Africa	Adolescents with negative QFT	RCT	BCG	Placebo	24			
Nemes	2018B	South Africa	Newborns of HIV infected mothers	RCT	MVA85A (TB candidate vaccine)	Placebo	12			
Pamra	1971	India	Asymptomatic persons with radiologically stable pulmonary TB lesions-smear/culture negative	RCT	Isoniazid	Placebo	72		✓	
Pape	1993	Haiti	Symptom free HIV seropositive	RCT	Isoniazid	Placebo	36		✓	✓
Pereira	2012	Brazil	7–14-year children with no BCG scar	cluster randomized trial	BCG	No intervention	63			
Quigley	2001	Zambia	HIV infected adults	RCT	Isoniazid	Placebo	36		✓	✓
Quigley	2001	Zambia	HIV infected adults	RCT	rifampicin plus pyrazinamide	Placebo	36			
Rangaka	2014	South Africa	HIV patients on ART	RCT	Isoniazid	Placebo	24		✓	✓
Rodrigues	2005	Brazil	school-aged children who had one BCG	cluster randomized trial	BCG	No intervention	-			
Ruan	2020	China	silicosis patients, with or without LTBI.	RCT	rifapentine +isoniazid (INH)	No intervention	37		✓	
Samandari	2015	Botswana	HIV infected adults	RCT	Isoniazid	Placebo	48		✓	✓
Samandari	2011	Botswana	HIV infected adults	RCT	Isoniazid	Placebo	57.6		✓	✓
Schechter	2006	Brazil	LTBI	RCT	Rifampin plus pyrazinamide	Rifapentine plus isoniazid	24	✓	✓	
Scriba	2021	south Africa	Adult without HIV and other comorbidities	RCT	Isoniazid +rifampicin	No intervention	180		✓	
Subramani	2015	south India	children aged <10 years	RCT	BCG	Placebo	180			
Sudfeld	2020	Tanzania	HIV infected adults on ART	RCT	vitamin D3 supplementation	Placebo	12			

Swaminathan	2012	India	HIV infected adults	RCT	Isoniazid	Ethambutol + isoniazid	36	✓	✓	✓
Swindells	2019	AANS	HIV infected adults	RCT	Isoniazid	Rifapentine + isoniazid	39.6	✓	✓	✓
Tait	2019	SKZ	18 to 50 years of age adults with LTBI	RCT	M72/AS01E (TB vaccine)	Placebo	36			
Tameris	2013	south Africa	infants without HIV who had received BCG	RCT	MVA85A (TB candidate vaccine)	Placebo	37			
Torre-Cisneros	2015	Spain	LTBI recipients of liver transplantation	RCT	Levofloxacin	Isoniazid	9	✓	✓	
Tuberculosis Research Centre	2013	South India	individuals irrespective of the tuberculin status	RCT	BCG	Placebo	180			
Vikrant	2005		recipients of haemodialysis and renal transplant.	RCT	isoniazid (INH)	Placebo	3.78		✓	
Villarino	2015	UCBCS	Children with latent tuberculosis infection.	RCT	Isoniazid+ Rifapentine	Isoniazid	33	✓	✓	
von Reyn	2010	Tanzania	HIV-positive residents with visible BCG scar	RCT	mycobacterium vaccae booster	Placebo	39.6			
Whalen	1997	Uganda	HIV patients with positive tuberculin skin tests	RCT	Isoniazid	Placebo	15		✓	✓
Whalen	1997	Uganda	HIV patients with positive tuberculin skin tests	RCT	Isoniazid plus rifampin	Placebo	15			
Whalen	1997	Uganda	HIV patients with positive tuberculin skin tests	RCT	Isoniazid plus rifampin plus pyrazinamide	Placebo	15			
Whalen*	1997	Uganda	HIV infected adults non-reactive to PPD	RCT	Isoniazid	Placebo	15			
Zar	2007	South Africa	children aged ≥8 weeks with HIV	RCT	Isoniazid	Placebo	5.7		✓	✓

SEM: South Africa, Ethiopia, and Mozambique; UMHB:US, Mexico, Haiti, Brazil; CFGHPRY: Czechoslovakia, Finland, German, Hungary, Poland, Romania, and Yugoslavia; ; UCBCS: United States, Canada, Brazil, China Spain; AANS: Africa, Asia, North America and South America; SKZ: south Africa, Kenya, Zambia; PLHIV: people living with HIV.

Suplementary figures

Suplementary Figure 1: Quality of included trials

study	D1	D2	D3	D4	D5	Overall
Agarwal 2004	+	+	+	+	+	+
Ahmed 2005	-	+	+	+	+	-
Apriani 2022	+	+	+	+	+	+
Barreto 2011	+	!	+	+	+	+
Bunyasi 2017	+	+	+	+	+	+
Bush 1965	+	+	+	+	+	+
Campa2017	+	+	+	+	+	+
Churchyard 2003	+	!	+	+	+	!
Churchyard 2014	+	+	+	+	+	+
Churchyard 2021	+	+	+	+	+	+
Comstock 1979	+	!	+	+	+	!
Comstock 1967	+	+	+	+	+	+
Comstock 1966	-	+	+	+	+	-
Comstock 1974	-	+	+	+	+	-
Comstock 1976	-	+	+	+	+	-
Cowie 1996	+	+	+	+	+	+
Crook 2016	+	+	+	+	+	+
Debre 1973	-	!	+	+	+	-
Dias de Oliveira 2020	+	+	+	+	+	+
Dorken 1984	!	+	+	+	+	!
Durovni 2013	+	+	+	+	+	+
Egsmose 1965	+	+	+	+	!	!
Falk 1978	+	+	+	+	+	+
Fitzgerald 2000	+	+	+	+	+	+
Frigati 2011	+	+	+	+	+	+
Gao 2018	+	!	+	+	+	!
Golub 2015	+	!	+	+	+	!
Gordin 2000	+	!	+	+	+	!
Gordin 1997	+	+	+	+	+	+
Gray 2014	+	+	+	+	+	+
Grzybowski 1976	-	-	+	+	+	-
Haller 1999	+	!	+	+	+	!
Halsey 1998	+	!	!	+	+	!
Hart 1977	+	+	+	+	+	+
Hawken 1997	+	+	+	+	+	+
Horwitz 1974	+	+	+	+	+	+
IUAT 1982	+	+	+	+	+	+
Jimenez Fuentes 2013	+	+	+	+	+	+
Katoch 2008	+	+	+	+	+	+
Khawcharoenporn 201	-	+	+	+	+	-
Kim 2015	+	+	+	+	+	+
LaCourse 2021	+	!	+	+	+	!
Luabeya 2015	-	+	+	+	+	-
Madhi 2011	+	+	+	+	+	+
Martison 2011	+	+	+	+	+	+
Menzies 2018	+	!	+	+	+	+
Mohammed 2007	+	+	+	+	+	+
Munseri 2020	+	+	+	+	+	+
Mwinga 1998	+	+	!	+	+	+
Naqvi 2006	+	+	+	+	+	+
Naqvi 2010	+	!	+	+	+	!
Nazareth 1971	+	+	+	+	+	+
Nazareth 1977	+	!	+	+	+	!
Ndiaye 2015	+	+	+	+	+	+
Nemes 2018 A	+	+	+	+	+	+
Nemes 2018 B	+	+	+	+	+	+
Pamra 1971	+	+	+	!	+	+
Pape 1993	+	+	+	+	+	+
Pereira 2012	+	+	+	+	+	+
Prevention Trial 1996	+	!	+	+	+	!
Quigley 2001	+	+	+	+	+	+
Rangaka 2014	+	+	+	+	+	+
Rodrigues 2005	+	+	+	+	+	+
Ruan 2020	+	+	+	+	+	+
Samandari 2015	+	!	+	+	+	!
Samandari 2011	+	+	!	+	+	!
Schechter 2006	+	+	+	+	+	+
Scriba 2021	+	!	+	+	+	!
Subramani 2015	+	+	+	+	+	+
Sudfeld 2020	+	+	+	+	+	+
Swaminathan 2012	+	!	+	+	+	!
Swindells 2019	+	!	+	+	+	!
Tait 2019	+	+	+	+	+	+
Tameris 2013	+	+	+	+	+	+
Torre-Cisneros 2015	+	!	+	+	+	-
TRC 1992	+	+	+	+	+	+
TRC 2013	+	+	+	+	+	+
Vikrant 2005	+	+	+	+	+	+
Villarino 2015	+	!	!	+	+	!
von Reyn 2010	+	+	+	+	+	+
Whalen 1997	+	+	+	+	+	+
Zar 2007	+	+	+	+	+	+

+	Low risk
!	some concerns
-	High-risk
D1:	Bias from randomisation process
D2:	deviations from intended interventions
D3:	Bias due to missing outcome data
D4:	Bias in measurement of the outcome
D5:	Bias in selection of the reported result

Supplementary Figure 2: Pair wise meta-analysis of preventive interventions.

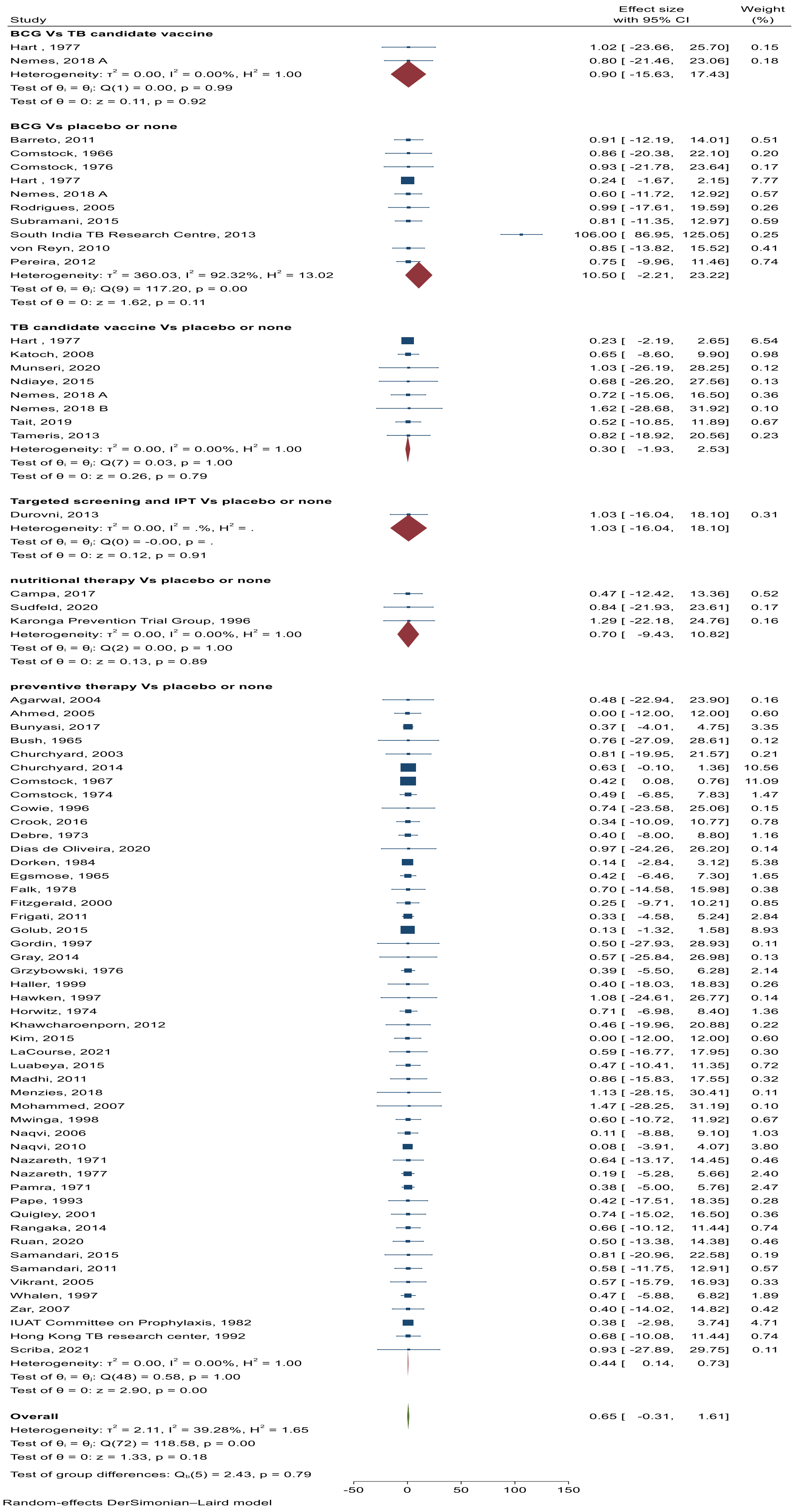

Supplementary Figure 3: Comparison based funnel plot of preventive interventions of TB incidence.

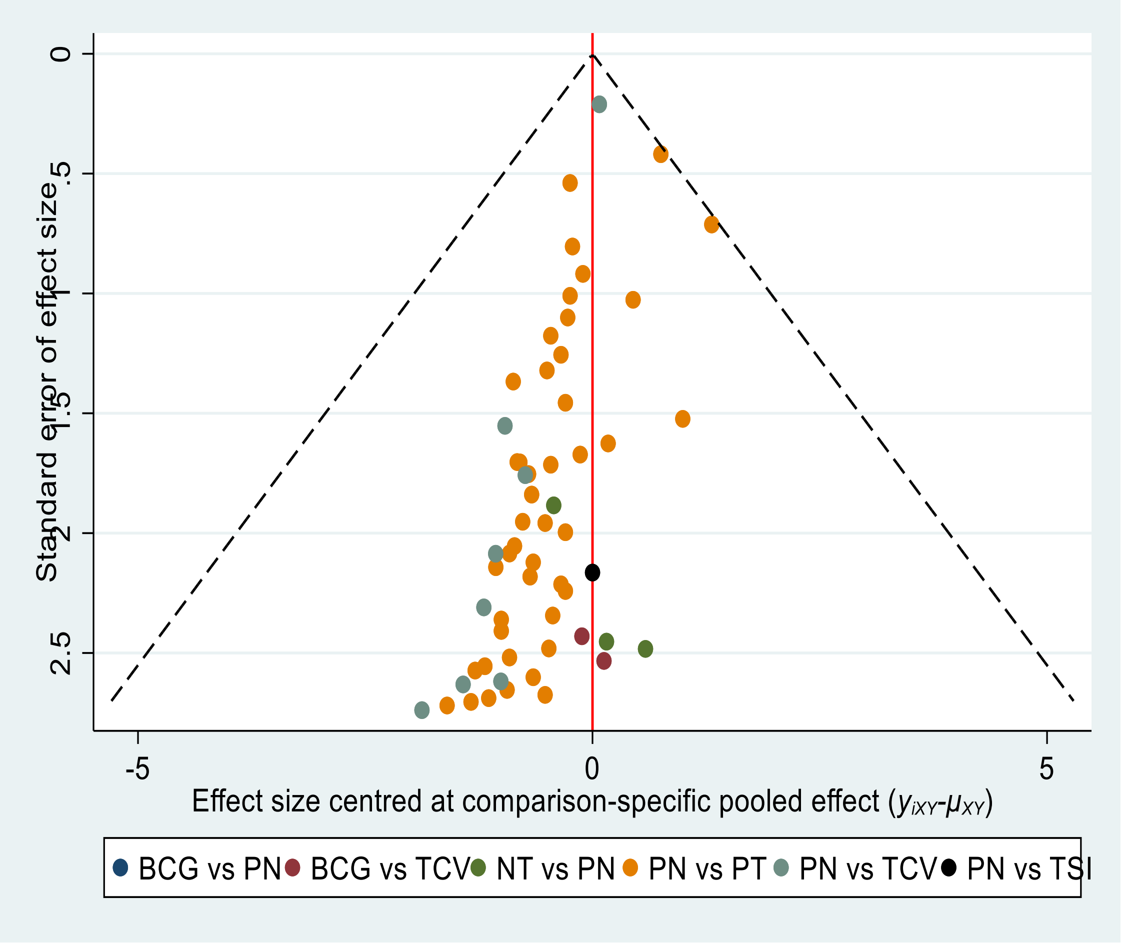

Supplementary Figure 4: Node splitting inconsistency test for preventive interventions.

(**Note**: D and E refers to BCG and TB candidate vaccines respectively).

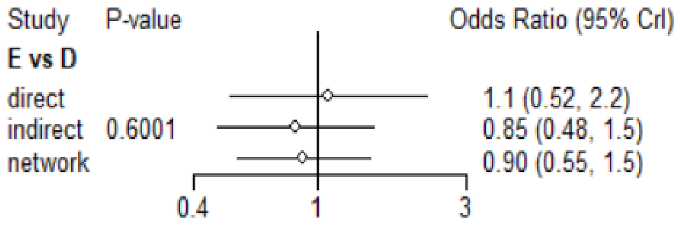

Supplementary Figure 5: Forest plot of relative effects for sensitivity analysis of preventive interventions excluding high risk and some concern studies.

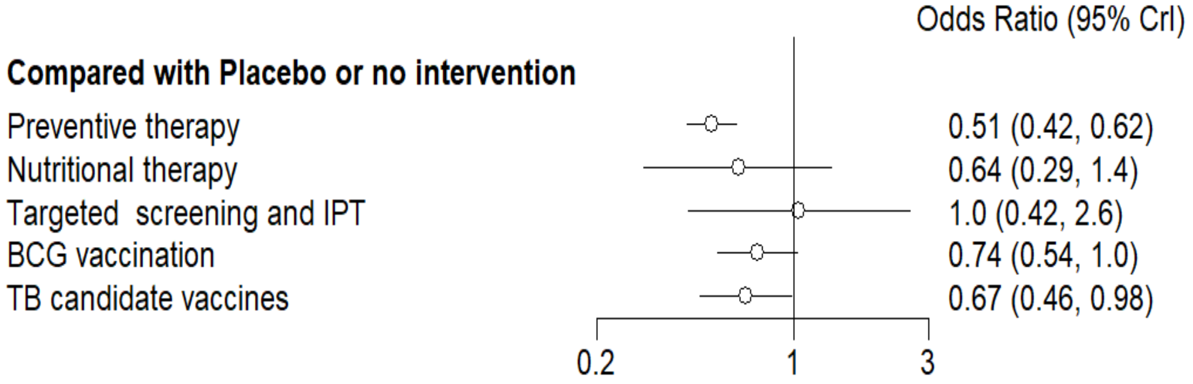

Supplementary Figure 6: Ranking probabilities of preventive interventions of TB excluding high risk and some concern studies.

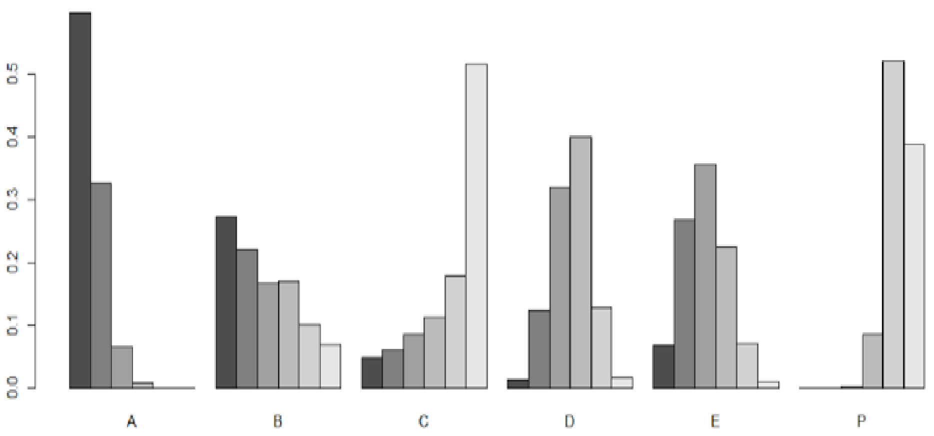

Note: The Y-axis indicates the probability of being the first the second and so on most likely effective intervention. The X-axis indicates the specific intervention that has been compared. Thus, “A”, “B”, “C”, “D”, “E” and “P” refer to preventive therapy, nutritional therapy, screening plus IPT, BCG vaccination, TB candidate vaccines and placebo or no intervention respectively.

Supplementary Figure 7: Node splitting inconsistency test for preventive treatments.

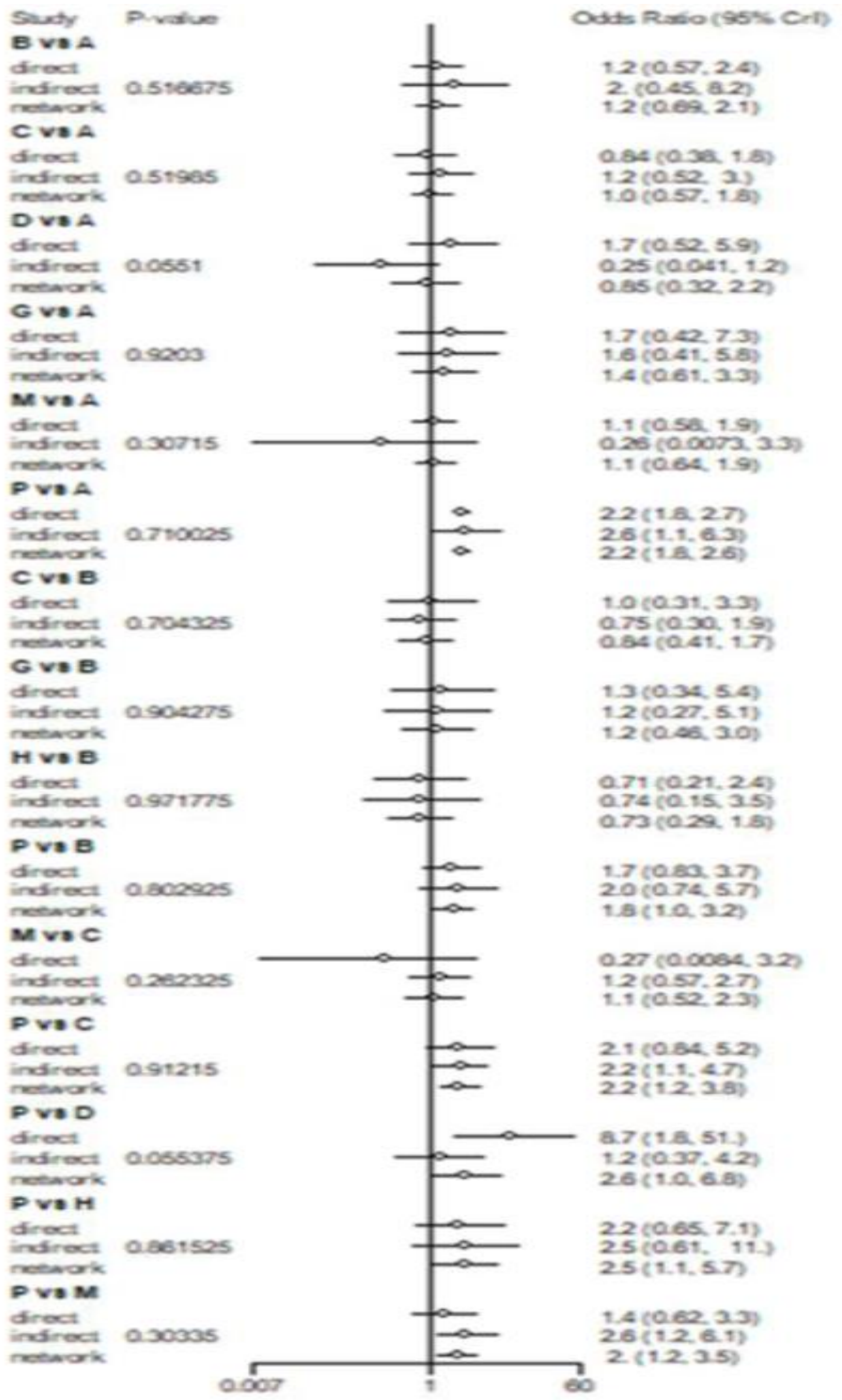

Note: "A", "B", "C", "D", "E", "F", "G", " H", " M", " N" , " O" and "P" represent isoniazid, isoniazid plus rifampicin, isoniazid plus rifapentine, isoniazid plus ethambutol, isoniazid plus streptomycin, isoniazid plus sulphadoxine-pyrimethamine, isoniazid plus rifampicin plus pyrazinamide, rifampicin, rifampicin plus pyrazinamide, levofloxacin, cotrimoxazole and placebo or no treatment respectively.

Supplementary Figure 8: Ranking probabilities of effectiveness of preventive treatments of TB.

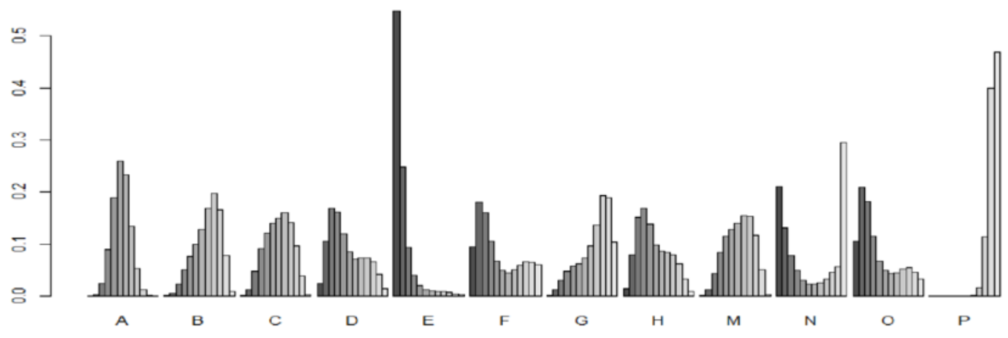

Note: "A", "B", "C", "D", "E", "F", "G", "H", "M", "N" , " O" and "P" represent isoniazid, isoniazid plus rifampicin, isoniazid plus rifapentine, isoniazid plus ethambutol, isoniazid plus streptomycin, isoniazid plus sulphadoxine-pyrimethamine, isoniazid plus rifampicin plus pyrazinamide, rifampicin, rifampicin plus pyrazinamide, levofloxacin, cotrimoxazole and placebo or no treatment respectively.

Supplementary Figure 9: Subgroup analysis of effective preventive treatments among PLHIV

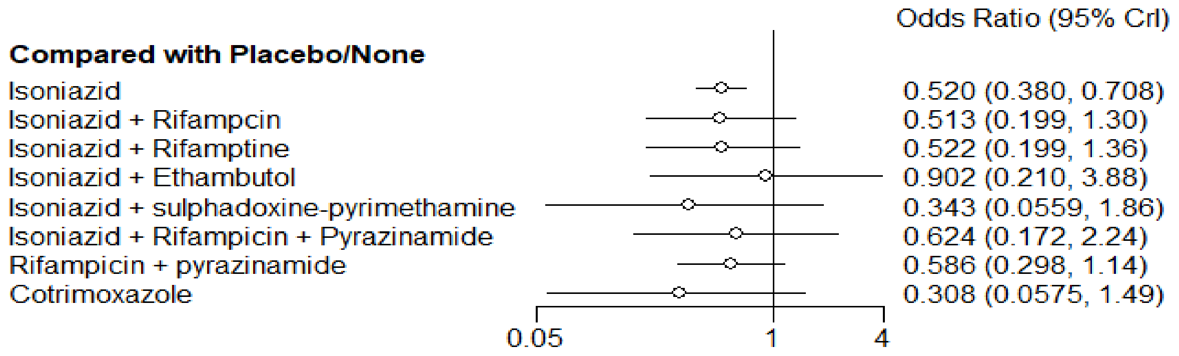

Supplement: Suplementary file [file mmc1.pdf]
